# Preparation of cobalt sulfide@reduced graphene oxide nanocomposites with outstanding electrochemical behavior for lithium-ion batteries†

**DOI:** 10.1039/d0ra01351j

**Published:** 2020-04-02

**Authors:** Junhai Wang, Yongxing Zhang, Jun Wang, Lvlv Gao, Zinan Jiang, Haibo Ren, Jiarui Huang

**Affiliations:** School of Material and Chemical Engineering, Chuzhou University Chuzhou 239000 P. R. China; Anhui Province Key Laboratory of Pollutant Sensitive Materials and Environmental Remediation, Huaibei Normal University Huaibei 235000 P. R. China zyx07157@mail.ustc.edu.cn; Key Laboratory of Functional Molecular Solids, Ministry of Education, Key Laboratory of Electrochemical Clean Energy of Anhui Higher Education Institutes, College of Chemistry and Materials Science, Anhui Normal University Wuhu 241002 P. R. China jrhuang@mail.ahnu.edu.cn

## Abstract

Cobalt sulfide@reduced graphene oxide composites were prepared through a simple solvothermal method. The cobalt sulfide@reduced graphene oxide composites are composed of cobalt sulfide nanoparticles uniformly attached on both sides of reduced graphene oxide. Some favorable electrochemical performances in specific capacity, cycling performance, and rate capability are achieved using the porous nanocomposites as an anode for lithium-ion batteries. In a half-cell, it exhibits a high specific capacity of 1253.9 mA h g^−1^ at 500 mA g^−1^ after 100 cycles. A full cell consists of the cobalt sulfide@reduced graphene oxide nanocomposite anode and a commercial LiCoO_2_ cathode, and is able to hold a high capacity of 574.7 mA h g^−1^ at 200 mA g^−1^ after 200 cycles. The reduced graphene oxide plays a key role in enhancing the electrical conductivity of the electrode materials; and it effectively prevents the cobalt sulfide nanoparticles from dropping off the electrode and buffers the volume variation during the discharge–charge process. The cobalt sulfide@reduced graphene oxide nanocomposites present great potential to be a promising anode material for lithium-ion batteries.

## Abstract 2

Cobalt sulfide@reduced graphene oxide nanocomposites obtained through a dipping and hydrothermal process, exhibit ascendant lithium-ion storage properties.

## Graphical abstract

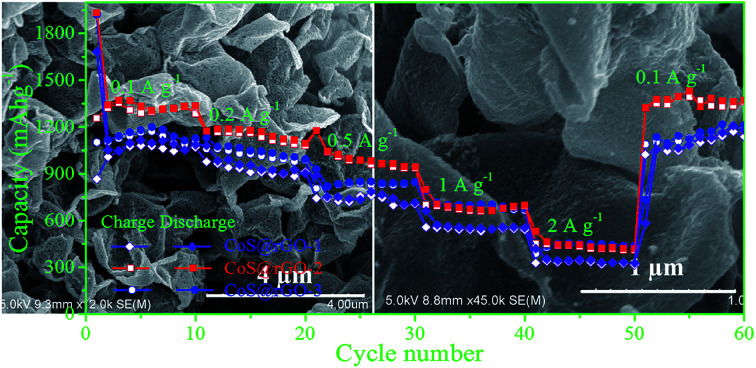

## Introduction

Nowadays, lithium-ion batteries (LIBs), as convenient and effective devices for the storage and conversion of energy, have extended their practical application in alternative energy sources, and hybrid electric vehicles, as well as other electrical equipment.^1–3^ The electrode materials largely determine some key electrochemical properties including its power density and cyclic-life. The present commercial anode, graphite with a low theoretical capacity of 372 mA h g^−1^, greatly hinders the application fields of LIBs.^4–6^ Thus, the search for an anode with a high capacity, high rate capability and good cycling stability becomes urgent for the extensive applications of LIBs.

Recently, various transition metal sulfides (MS_*x*_) such as FeS_*x*_,^7^ NiS_*x*_,^8,9^ MnS_*x*_,^10^ MoS_2_,^11,12^ SnS_2_,^13^ CuS,^14,15^ ZnS^16^ have been fabricated and served as anode materials for LIBs due to their high theoretical capacities, low cost and abundant raw materials. Cobalt sulfide (CoS_*x*_) has been considered as an alternative anode material because of its high theoretical specific capacity of 590 mA h g^−1^ and superior electrochemical performance.^17^ Unfortunately, the pure CoS anode suffers from the low rate capability and a poor cycling stability, which were caused by the low electric conductivity, huge-structural change, and easy pulverization of the CoS anode material.^17–20^ Therefore, the tremendous efforts have been paid to improving the conductivity of anode materials and the diffusion of lithium ions/electrons.

To address the above problems, many attentions have been paid to the design of the CoS-based composites with nanostructures. The nanostructured CoS composites with an improved structural stability and electronic conductivity could be fabricated by incorporation of the CoS nanoparticles (NPs) with various carbonaceous materials such as amorphous carbon,^21^ carbon nanotubes,^22^ carbon nanofibers.^23^ These days, graphene, one of the most sparking carbonaceous materials, has attracted widespread attention because of its superior physical and chemical merits such as huge specific surface area, exceptional electrical conductivity and structural stability.^24–26^ It is formed by the hybridization of sp^2^ carbon atom, shaped by the 2D single atom layer. The graphene has been thought as a desirable conductive substrate to incorporate the CoS NPs.^27–29^ For example, Tan *et al.* synthesized CoS nanofibers (NFs) anchored on reduced graphene oxide (rGO) *via* combination of hydrothermal with sulfidation process. The as prepared CoS NFs–rGO electrodes delivered the discharge a capacity of 939 mA h g^−1^ after the 100th cycle at 100 mA g^−1^ with coulombic efficiency above 98%.^30^ Zhu *et al.* prepared CoS_*x*_/rGO nanocomposite *via* a facile hydrothermal method with CoS_*x*_ NPs uniformly embedded in rGO. When utilized in LIBs, the composite demonstrated a high discharge capacity of 796 mA h g^−1^ after 50 cycles at 100 mA g^−1^.^31^ Lu *et al.* reported Co_1−*x*_S hollow spheres formed by *in situ* growth on reduced graphene oxide layers. When evaluated as an anode material for LIBs, it delivers a specific capacity of 969.8 mA h g^−1^ with a high coulombic efficiency of 96.49% after 90 cycles at 50 mA g^−1^.^32^ Compared with the pure CoS anodes, improvements in the specific capacity, cycling life and rate performance were achieved. Nevertheless, most graphene-based nanocomposites were obtained through a simple hydrothermal method, mixing the reactant with graphene oxide in a Teflon-lined autoclave. The resulting active materials obtained by this method cannot fully anchor on the surface of the rGO and often fall off easily, leading to a poor electrochemical property.^25,27,32,33^ Furthermore, the loading amount of CoS on rGO would also greatly affect the Li-storage performances of the nanocomposites. To seek for the desirable electrochemical behaviors for LIBs, it is important to develop a CoS@rGO nanocomposite with various CoS loadings, in which all CoS could anchor on the surface of rGO densely and tightly.

In this study, CoS@rGO nanocomposites with a 3D nanostructure were synthesized through a simple hydrothermal method. The composites consist of numerous CoS NPs and the rGO. The functional groups on the surfaces of rGO capture the Co ions *via* an electrostatic reaction to form CoS NPs uniformly distributing on the surfaces of the rGO. Employed as an anode, the CoS@rGO nanocomposites show outstanding electrochemical behaviors for LIBs.

## Experimental

All chemical reagents in this study were at the analytical level. The preparation of graphene oxide and 3D rGO is described in the ESI.†

### Preparation of CoS@rGO

Synthesis of CoS@rGO 0.8 g of CH_3_CSNH_2_ and various amount of CoCl_2_·6H_2_O (Sinopharm Chemical Reagent Co., Ltd) were dissolved in 14 mL of DMF/water mixture solvent (1 : 1 v/v). The mixed precursor solution was stirred to form a transparent solution at room temperature using a magnetic stirrer. Then, a columnar rGO was soaked in the solution for 2 days. After that, the solution system was transferred into a Teflon-lined stainless steel autoclave and maintained at 200 °C for 24 h. When it cooled down to room temperature, the product was taken out and rinsed for several times with deionized water and absolute ethanol, respectively. At last, the product was vacuum-dried at 60 °C for 10 h. The amounts of CoCl_2_·6H_2_O for the preparation of CoS@rGO-1, CoS@rGO-2 and CoS@rGO-3 were 0.4, 0.5 and 0.6 g, respectively.

### Material characterizations

The as-prepared samples were characterized using X-ray diffraction (Shimadzu XRD-6000, high-intensity Cu Kα radiation with a characteristic wavelength of 1.54178 Å), transmission electron microscopy (TEM, Hitachi H-800, operated at acceleration voltage of 200 kV), field emission scanning electron microscopy (FE-SEM, Hitachi S-4800, operated at 5 kV) with energy dispersive spectroscopy (EDS), X-ray photoelectron spectroscopy (XPS) (ESCALAB 250), Brunauer–Emmett–Teller (BET) nitrogen adsorption–desorption (Nova 2000E), and Raman spectroscopy (Bruker-Senterra, wavelength 532 nm). A thermogravimetric analysis (Setaram Labsys Evo SDT Q600) was conducted to determine the rGO content of the composites.

### Electrochemical measurements

For a half-cell, the electrochemical tests of the as-prepared CoS@rGO nanocomposites were conducted in a CR2032 coin-kind cell using a polypropylene separator (Celgard 2400), and the lithium plate acted as a counter/reference electrode. The active materials mixed with a sodium carboxymethyl cellulose binder, styrene butadiene rubber, and carbon black (mass ratio of 7 : 1.5 : 0.5 : 1) in water to form a slurry. The uniform slurry was then cast onto the surface of a copper foil and dried at 80 °C for 12 h. The electrolyte consisted of 1 M LiPF_6_ mixed with ethylene carbonate (EC) and diethyl carbonate (DEC), with a volume ratio of 1 : 1. The mass loading of the electrode and the active mass are *ca.* 1.08 and 0.76 mg, respectively. The assembly process of the cells was carried out in a glove box (Mikrouna, Super (1220/750/900)) filled with Ar gas, with oxygen and water concentrations below 0.1 ppm and 1 ppm, respectively. For a full-cell, a commercial LiCoO_2_ was used as the counter/reference electrode using a polypropylene separator (Celgard 2400). The commercial LiCoO_2_ mixed with a binder polyvinylidene fluoride (PVDF) and carbon black (mass ratio of 8 : 1 : 1) in *N*-methyl-2-pyrrolidone (NMP) solvent to form a slurry. The uniform slurry was then cast onto the surface of an aluminum foil and dried at 80 °C for 12 h. The mass loading of the electrode and the active mass are *ca.* 10.01 and 8.01 mg, respectively. The ratio of cathode and anode active materials is *ca.* 10.54. For a half-cell, a Neware battery tester (Shenzhen Neware Technology Co., Ltd) with a voltage ranging from 0.01–3.0 V was used to monitor the discharge/charge behavior. For the full-cell, the voltage range was 1.5–3.9 V. An electrochemical workstation (CHI-660E, Shanghai Chenhua Instruments Co., Ltd) was used to record the cyclic voltammetry (CV) performance at a scanning rate of 0.1 mV s^−1^. The data of the electrochemical impedance spectrum (EIS) was obtained from 0.01 Hz to 100 kHz.

## Results and discussion

### Structural and morphological characterization

**Fig. 1:**
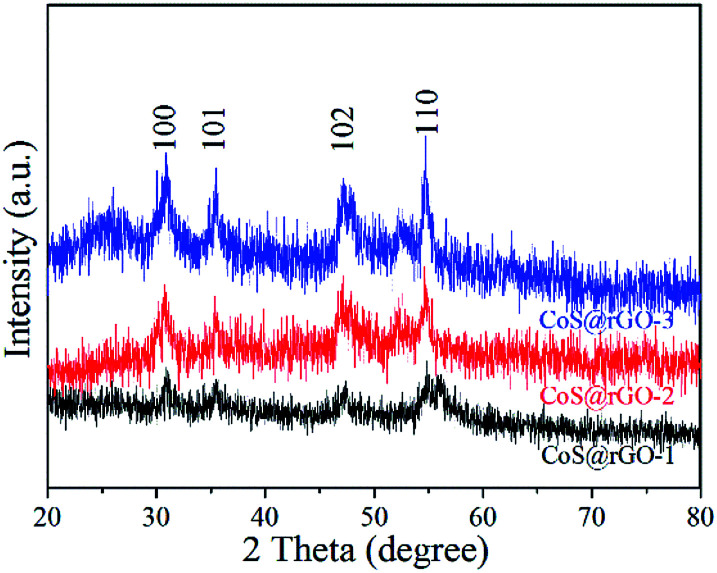
XRD pattern of three CoS@rGO samples.

The crystal structure of the CoS@rGO samples were determined by XRD as shown in Fig. 1. Three XRD patterns all exhibit four typical diffraction peaks at around 30.8°, 35.3°, 47.1°, and 54.7°, which are ascribed to the (100), (101), (102) and (110) crystal planes of hexagonal CoS, respectively. The result is consistent with the standard card of CoS (JCPDS no. 75-0605). It should be noted that the peak of rGO at the location of 20–30° cannot be detected, in comparison with the XRD patterns of the 3D rGO made freshly, as exhibited in Fig. S1.† A possible reason can explain it that the agglomeration of the rGO can be effectively prevented due to CoS NPs closely anchored on the surface of the rGO.^34,35^ No other obvious diffraction peaks are found in Fig. 1, indicating that the composite has a high purity.

**Fig. 2:**
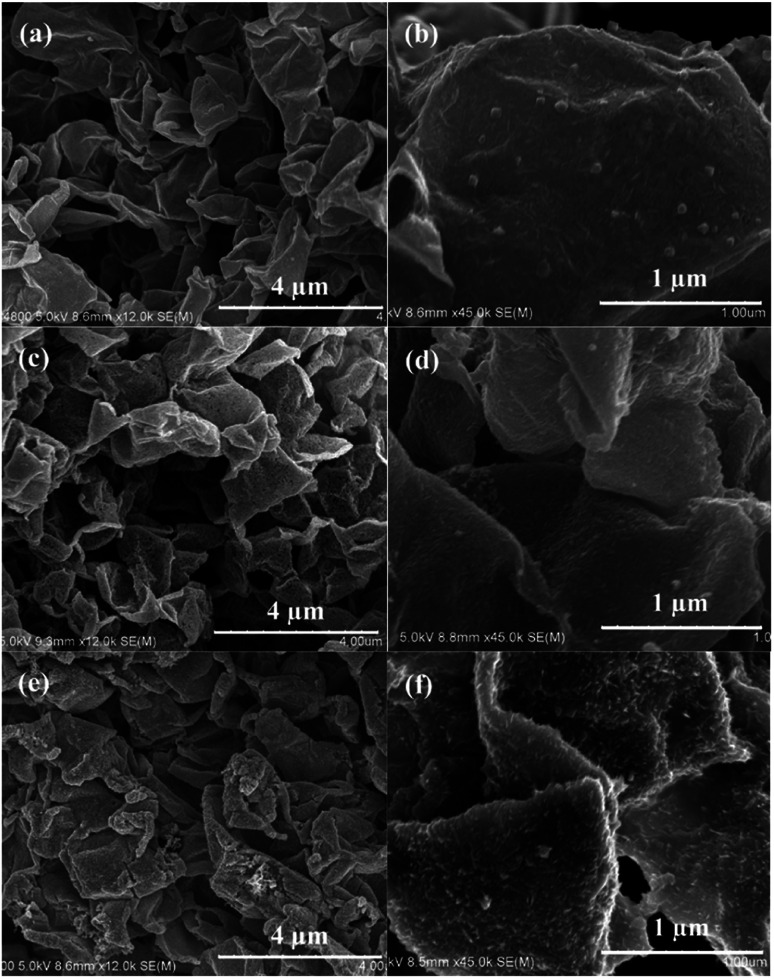
SEM images of (a and b) CoS@rGO-1, (c and d) CoS@rGO-2 and (e and f) CoS@rGO-3 nanocomposites.

The FE-SEM images of the samples (CoS@rGO-1, CoS@rGO-2 and CoS@rGO-3) are presented in Fig. 2. From the low-magnification images (Fig. 2a, c, and e) of the samples, a sheet-like structure can be seen, which consists of numerous crumpled rGO-based nanosheets. Compared to the SEM image of bare rGO (Fig. S2a†), the high magnification images (Fig. 2b, d, and f) demonstrate that there are a large number of nanoparticles on the surface of rGO. Further observation reveals that the amount of agglomerated nanoparticles anchored on the rGO nanosheets gradually increased with the increase of the CoS mass ratio.

**Fig. 3:**
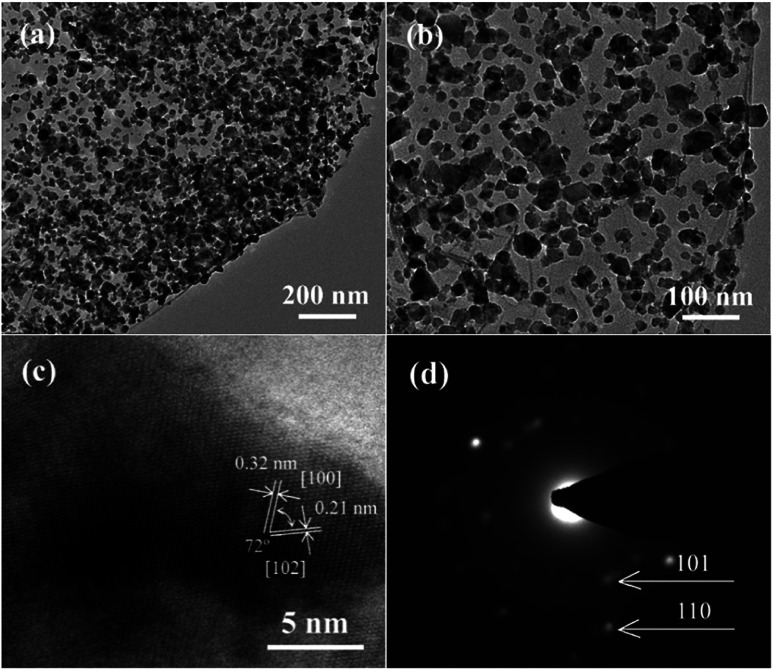
(a and b) TEM images, (c) HRTEM image and (d) SAED pattern of CoS@rGO-1 nanocomposites.

More detailed information on the morphology and structure of CoS@rGO-1 could be obtained using TEM and high-resolution TEM (HRTEM). The TEM images of the CoS@rGO-1 composite are presented in Fig. 3a and b. It can be seen that the sample possesses a wrinkled thin-sheet structure. Compared to the TEM image of bare rGO (Fig. S2b†), the high resolution TEM image (Fig. 3b) reveals that the rGO sheet was uniformly covered by a large number of CoS NPs, with sizes no larger than 50 nm. Lattices fringes are clearly observed in Fig. 3c, the HRTEM image. Two interplanar distances are 0.32 nm and 0.21 nm, which well correspond to the (100) and (102) lattice planes of hexagonal CoS, respectively. Fig. 3d displays the selected-area electron diffraction (SAED) pattern, from which two Debye–Scherrer rings are distinct and visible. And they conform to the (101) and (110) crystallographic directions of hexagonal CoS, which well correspond to the XRD results (Fig. 1).

**Fig. 4:**
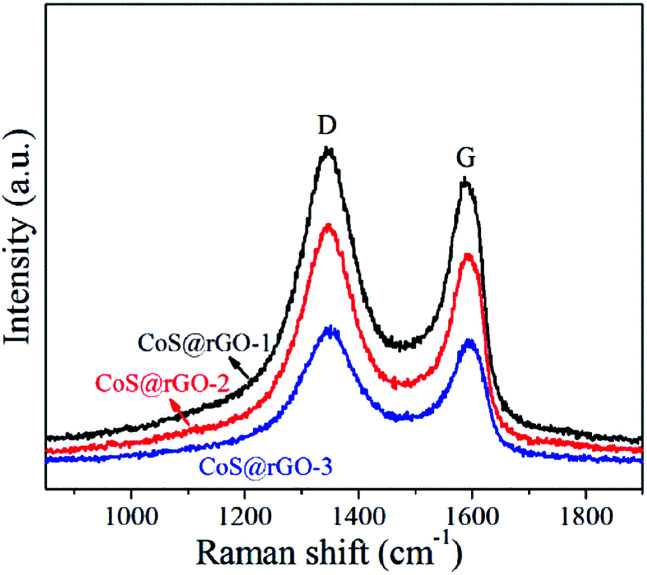
Raman spectra of three CoS@rGO samples

Raman spectroscopy was applied to investigate the structure of carbon materials in details. And Raman spectra of the CoS@rGO and GO samples are displayed in Fig. 4 and S3,† respectively. From the Raman spectra, two characteristic peaks are clearly discovered. They locate at *ca.* 1347 and 1588 cm^−1^, which can be respectively ascribed to the D and G bands of rGO. Furthermore, 2D and D + G peaks not shown here should locate at approximately 2694 and 2935 cm^−1^, respectively.^36^ The formation of the D and G bands results from the disorder and defects in the graphene layers, and sp^2^-bonded carbon atoms in the hexagonal lattice, respectively. For carbon-related materials, the intensity ratio (*I*_D_/*I*_G_) of the D and G bands represents the defects and degree of graphitization.^37^*I*_D_/*I*_G_ of the CoS@rGO-1, CoS@rGO-2, CoS@rGO-3, and GO are 1.12, 1.16, 1.22, and 0.917, respectively. This clearly indicates that most of the oxygen-containing functional groups on graphene oxide have been reduced in the CoS@rGO composites.^36^ Furthermore, the increase of the intensity ratio (*I*_D_/*I*_G_) may also be due to due to the widely separated rGO layers promoted by the intercalation of CoS NPs.^38^

**Fig. 5:**
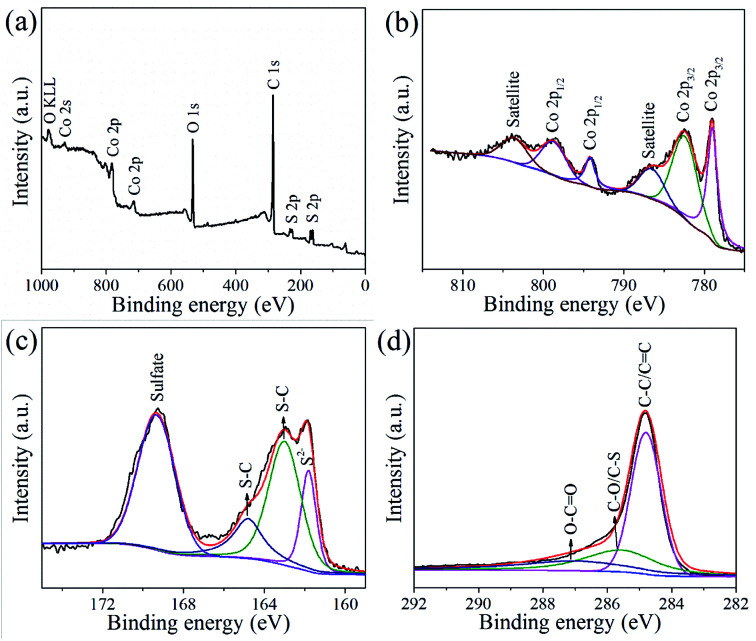
XPS spectra of CoS@rGO-2 sample: (a) survey spectrum, (b) Co 2p spectrum, (c) S 2p spectrum, and (d) C 1s spectrum.

XPS analysis was used to determine the valence state and elemental composition in the CoS@rGO nanocomposites. From the survey spectrum shown in Fig. 5a, the nanocomposite is comprised of the elements of Co, S and C, which is consistent with the results of the XRD and HRTEM. As shown in Fig. 5b, two peaks at 785.5 and 802.8 eV agree with satellite peaks of Co^2+^.^39^ The peaks at 778.5 and 793.7 eV are the Co 2p_3/2_ and 2p_1/2_ of cobalt sulfides.^40^ The binding energy at 780.6 and 796.3 eV are consistent with Co 2p_3/2_ and 2p_1/2_ with the splitting value over 15 eV demonstrating the coexistence of Co^3+^ and Co^2+^.^41,42^ The higher state of Co^3+^ would provide extra capacity for LIBs.^43^ The S 2p spectrum as shown in Fig. 5c can be deconvoluted into one peak at 169.4 eV typifying the oxidized-S, two peak at 164.8 and 163.0 eV corresponding to S–C bond and one peak at 161.8 eV indicating CoS.^32,44–47^ In addition, Fig. 5d exhibits the high-resolution C 1s spectra of the nanocomposites. The strong peak at 284.6 eV is originated from the C–C/C=C bonds. Also, two weak peaks at 288.5 eV and 286.3 eV are detected, coming from the O–C=O and C–O/C–S bonds, respectively.^48^ Obviously, the intensity of the C–C/C=C bonds are much stronger than that of O–C=O and C–O/C–S bonds. This result proves that the overwhelming majority of GO were reduced and transformed into the rGO *via* the hydrothermal process. The presence of C–S bond further confirms the formation of tight bonding between the C atoms and S atoms, which demonstrates that the CoS particles tightly attached on the surface of the rGO.^25^

The content of rGO in the CoS@rGO nanocomposite was calculated based on the TGA results, which were performed at the temperature range of room temperature to 650 °C in air, as shown in Fig. S4 (see ESI for detail).† It can be seen that the weight of the composite decreases with the increase of temperature. A slight decrease in the weight occurred before 290 °C, which may be due to the release of a volatile gas and water. The second weight loss observed from 290 to 410 °C is ascribed to the combustion of rGO.^49^ The third weight loss from 410 to 650 °C is mainly due to the decomposition by oxidation of CoS into Co_3_O_4_.^50^ As we know that the TGA data cannot be so clearly separated to rGO and CoS formation. The carbon contents within the CoS@rGO-1, CoS@rGO-2 and CoS@rGO-3 samples are approximately 4.5 ± 0.3 wt%, 3.6 ± 0.2 wt% and 3.1 ± 0.2 wt%, respectively.

For the investigation of the porosity texture of the CoS@rGO nanocomposites, the nitrogen adsorption–desorption isotherms and pore size distributions (inset) were performed in Fig. S5.† A hysteresis loop appears in the isotherms of three samples. This strongly indicates some mesopores existing in the nanocomposites. The specific surface areas were calculated as 57.6, 34.4 and 18.3 m^2^ g^−1^ for the CoS@rGO-1, CoS@rGO-2 and CoS@rGO-3 samples, respectively, in terms of a Brunauer–Emmett–Teller (BET) model. Obviously, the specific surface area of the samples slightly decreases as the load of the CoS NPs increases. The insets of the figures show the distributions of the pore sizes ranging from 1 to 22 nm. The pore sizes for the CoS@rGO-1, CoS@rGO-2 and CoS@rGO-3 respectively average 2.4, 2.1 and 1.9 nm. The as prepared CoS@rGO nanocomposites with high specific surface area and sandwich structure would present some exceptional performances in the application of LIBs.

### Electrochemical properties

**Fig. 6:**
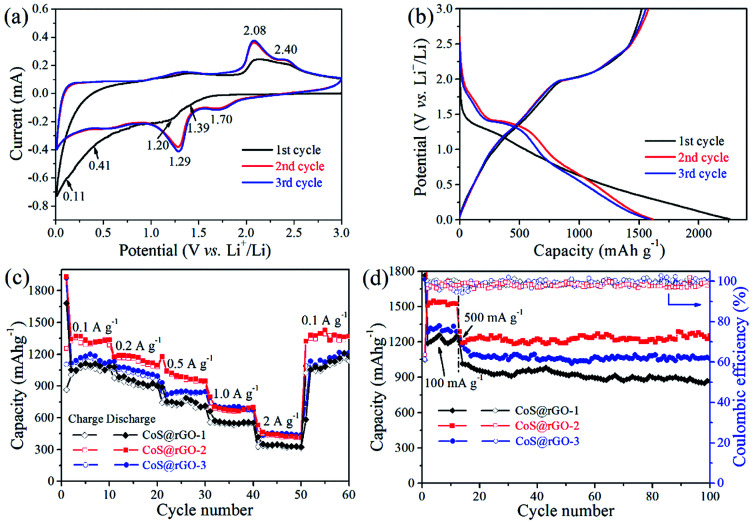
(a) Initial three cyclic voltammetry curves, (b) galvanostatic discharge/charge profiles of CoS@rGO-2 anode at the current density of 100 mA g^−1^, (c) rate performance of CoS@rGO composites at various current densities, and (d) cycling performance of CoS@rGO composites at the current density of 500 mA g^−1^.

The electrochemical properties of the electrodes were studied using cyclic voltammograms (CV) tests. Fig. 6a shows the initial three CV curves of the CoS@rGO-2 nanocomposite anode. In the first cycle, a slight peak at ∼1.39 V can be assigned to the Li insertion reaction: CoS + *x*Li^+^ + *x*e^−^ → Li_*x*_CoS.^19,33,51^ Another slight peak at ∼1.20 V corresponds to the reduction reaction: (2 − *x*)Li^+^ + Li_*x*_CoS + (2 − *x*)e^−^ → Co + Li_2_S.^19,33,51^ In the next two cycles, two peaks positively shift to ∼1.70 V and ∼1.29 V, respectively. The weak broad peak at *ca.* 0.41 V is assigned to the formation of the solid electrolyte interface (SEI) film which disappear in the following two cycles. The prominent peak at ∼0.11 V is attributed to the insertion of lithium into the rGO nanosheets and the lattice of carbon: *y*C + *x*Li^+^ + *x*e^−^ → Li_*x*_C_*y*_.^52,53^ In the anodic process, two apparent peaks at ∼2.08 and ∼2.40 V can be identified as the reverse reaction to form Li_*x*_CoS and CoS, respectively. The CV curves are overlapped well in the second and third cycles indicating the good electrochemical reversibility of the electrode.

The discharge and charge profiles the CoS@rGO-2 nanocomposites are shown in Fig. 6b. The discharge profiles reveal a plateau at ∼1.26 V in the first cycle, which elevates to ∼1.41 V in the following cycles. (The plateau at ∼0.45 V is not apparent corresponding to the unapparent peak of SEI film formation.^33^ The obvious plateau at ∼0.12 V is ascribed to the lithium ion insertion of carbon.) In the charge process, two plateaus at ∼2.0 and ∼2.4 V are consistent with the anodic peaks in CV curves. In the first discharge and charge curves, the CoS@rGO-2 composite delivers specific capacities of 2262.0 and 1521.9 mA h g^−1^, respectively. And the coulombic efficiency is calculated as 67.3%, which mainly due to the SEI layer formed on the surface of the electrode. Besides, in the first lithiation process, some lithium ions were consumed because of the defects in the nanocomposite.^54^ In the second and third cycles, the specific discharge capacities of the CoS@rGO-2 composites declined to 1613.4 and 1589.2 mA h g^−1^, respectively. The coulombic efficiency of the second cycle increased to 97.8%, and it maintained *ca.* 97.9% in the third cycle, proving the good electrochemical cycling reversibility.

The rate performance of the CoS@rGO-1, CoS@rGO-2, CoS@rGO-3 were presented in Fig. 6c. The CoS@rGO-2 electrode obviously showed the best rate capability among these electrode materials and had reversible specific capacities of 1435.8, 1244.9, 1109.3, and 892.1 mA h g^−1^ at the current densities of 0.1, 0.2, 0.5, and 1.0 A g^−1^, respectively. The CoS@rGO-2 electrode delivered a high capacity of 668.5 mA h g^−1^ even at 2.0 A g^−1^. For the CoS@rGO-1, CoS@rGO-2 and CoS@rGO-3 electrodes, the specific capacities could respectively reach to 1305.8, 1462.6 and 1335.1 mA h g^−1^, when the current density returns to 0.1 A g^−1^. Furthermore, the CoS@rGO nanocomposite electrodes present a much better rate capacity than those of cobalt sulfide-based composites such as CoS NFs–rGO,^30^ CoS_*x*_/rGO nanocomposite^31^ and Co_1−*x*_S hollow spheres/rGO.^32^

The cycling performance of the CoS@rGO composites was investigated when a high current density was set as 0.5 A g^−1^. Observed from Fig. 6d, after 100 cycles, the nanocomposites of the CoS@rGO-1, CoS@rGO-2 and CoS@rGO-3 electrodes still hold corresponding specific capacities of 868.1, 1253.9 and 1056.6 mA h g^−1^ with the coulombic efficiency of 99.2%, 98.9% and 99.9%, respectively. The theoretical capacity of CoS@rGO-2 can be calculated to be ∼596.6 mA h g^−1^ (CoS contribution (capacity_CoS_ = 590 × 96.4%) + rGO contribution (capacity_rGO_ = 774 × 3.6%)). The capacity of the CoS@rGO-2 electrode is much higher than the theoretical value, which may be attributed to an excellent synergistic effect between CoS NPs and rGO.^48^ In comparison with the CoS-based electrodes recent reported, the CoS@rGO nanocomposite electrodes exhibit superior electrochemical performance than those of CoS NPs,^18^ lantern-like CoS,^55^ CoS/CNTs hybrid,^22^ CoS nanosheets/rGO foams,^25^ cobalt sulfides/rGO composite,^27,28^ CoS NFs–rGO,^30^ CoS_*x*_/rGO nanocomposite,^31^ Co_1−*x*_S hollow spheres/rGO,^32^ CoS_2_/rGO composites,^38^ and other CoS/rGO composites,^33,56,57^ which have been listed in Table S1 (ESI).† The superior electrochemical properties of CoS@rGO can be well-explained by two aspects: (i) rGO sheets can mitigate the volume variation of the CoS NPs during the lithium reaction; (ii) layered nanoarchitecture facilitates the electrolyte access, as well as enlarges the contact area between the CoS@rGO nanocomposites and the electrolyte, resulting in improved transport kinetics of the lithium ions. Therefore, the as-prepared nanocomposites can be potentially considered as a suitable electrode material in the future application of lithium-ion battery. Notably, the CoS@rGO-2 electrode exhibits the highest discharge capacity (1253.9 mA h g^−1^) among three nanocomposite electrodes after 100 cycles. And the suitable loading of active materials (CoS NPs) in the nanocomposites could account for the highest discharge capacity of the CoS@rGO-2 electrode. Since the theory capacity of CoS is higher than that of the rGO, the capacity of the CoS@rGO increases with the increase of the amount of the CoS within the composites. As a result, the specific capacity of the CoS@rGO-2 is higher than that of CoS@rGO-1. However, when there are too many CoS NPs anchored on the rGO, they could easily detached from rGO during the charge–discharge process, resulting a poor capacity.^58^ The detachment of the CoS NPs from the rGO can be observed from the SEM and TEM images (Fig. S7†) of the CoS@rGO-3 electrode after 100 cycles at 0.5 A g^−1^. Therefore, the specific capacity of the CoS@rGO-3 is lower than that of CoS@rGO-2.

**Fig. 7:**
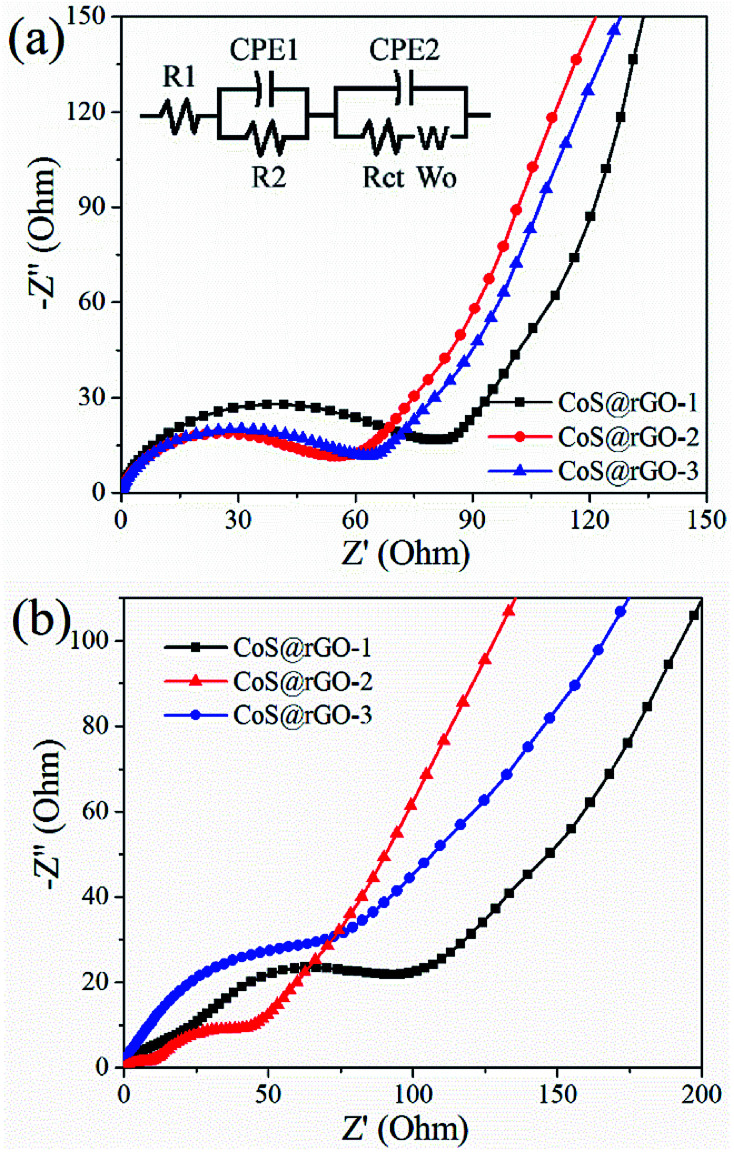
EIS impedance spectra of CoS@rGO electrodes (a) before cycling test and (b) after 100 cycles. Inset is the equivalent electrical circuit model.

**Fig. 8:**
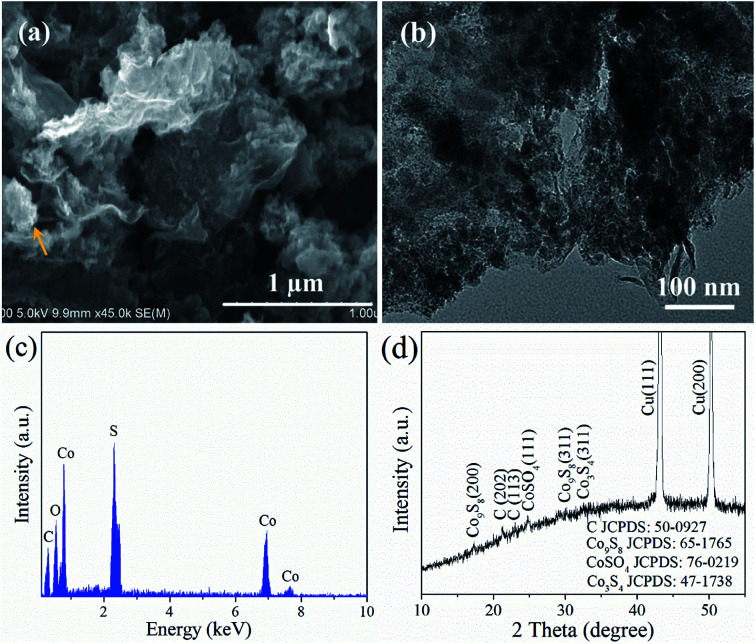
(a) SEM image, (b) TEM image, (c) EDS, and (d) XRD pattern of CoS@rGO-2 electrode after 100 cycles at a current density of 500 mA g^−1^.

The Nyquist plots of the CoS@rGO nanocomposite electrodes before cycling test and after 100 cycles were presented in Fig. 7. The experimental data was fitted by an equivalent circuit, as exhibited in Fig. 7a (inset). In this model, the internal resistance of the battery was denoted by *R*_s_. The resistance and constant phase element for the SEI film can be respectively expressed by *R*_f_ and CPE1. And for electrode/electrolyte interface, the charge-transfer resistance and constant phase element can be represented by *R*_ct_ and CPE2, respectively. In addition, the Warburg impedance produced in the process of the lithium-diffusion can be referred as *W*_o_.^59^ The *R*_ct_ of the CoS@rGO-1, CoS@rGO-2 and CoS@rGO-3 electrodes before cycling test are 80.7, 56.6 and 61.3 Ω, respectively, which can be revealed by the fitting results. After 100 cycles, *R*_ct_ of these electrodes respectively decreases to 53.0, 20.1 and 40.5 Ω. This may be due to the obvious pulverization of CoS NPs on the surface of rGO during the cycling process. The pulverization of CoS NPs can be observed from the TEM image (Fig. 8b) of the CoS@rGO-2 electrode after 100 cycles at 0.5 A g^−1^. Among these CoS@rGO nanocomposite electrodes before cycling test and after 100 cycles, the *R*_ct_ of CoS@rGO-2 electrode is the smallest, for CoS NPs anchored on the rGO in the composites contribute to the small *R*_ct_ value. Moreover, the *R*_ct_ of the CoS@rGO-3 electrode is higher than that of the CoS@rGO-2 electrode, which may be due to the slight detachment of active material CoS from the surface of rGO. The slight detachment of the CoS NPs marked with an arrow can be observed from the SEM image (Fig. 8a) of the CoS@rGO-2 electrode after 100 cycles at 0.5 A g^−1^. The straight line in the low-frequency region is associated with Warburg behaviour where the lithium-ion diffusion occurs in the nanostructure. This clearly indicates that the unique porous structure is quite advantageous for improving the speed of charge transfer at the interface, and reducing the overall resistance of the battery, thus leading to excellent electrochemical performances.

The morphology and composition of the CoS@rGO nanocomposites were investigated by SEM, TEM, EDS, and XRD after the composite electrode was tested at 0.5 A g^−1^ for 10 or 100 cycles. Fig. S8† shows the TEM image of the composites after 10 cycles. It can be found that the CoS NPs still anchor on the surface of rGO and most NPs have no obvious change in size. Fig. 8a and b show the SEM and TEM images of the composites after 100 cycles. The rGO still maintains its original nanosheet-like morphology, and the vast majority of ultra-small NPs are uniformly attached on the rGO. It can be concluded that the CoS NPs become small after 100 cycles, and most of them still firmly anchor on the surface of the rGO, demonstrating the outstanding stability of the nanocomposites. In addition, the presence of the rGO facilitates the good distribution of the ultra-small NPs during the cycling process. The EDS analysis shown in Fig. 8c indicates that the composites still consist of the original elements of Co, S and C after cycling. Fig. 8d presents the XRD pattern of the composites after 100 cycles and indicates the presence of cubic Co_3_S_4_, cubic Co_9_S_8_, orthorhombic CoSO_4_, and rhombohedral carbon in the final electrode material. This is consistent with the result of the EDS analysis after cycling. Moreover, Fig. S6† shows the XPS spectra of the nanocomposite electrode after 100 cycles at 0.1 A g^−1^. This clearly shows the presence of Co, S and C in the composites after 100 cycles. A small part of S has been oxidized to some higher valence states, and after the cycles, the composite consists of S^2−^, S_2_^2−^, SO_3_^2−^, and SO_4_^2−^.

**Fig. 9:**
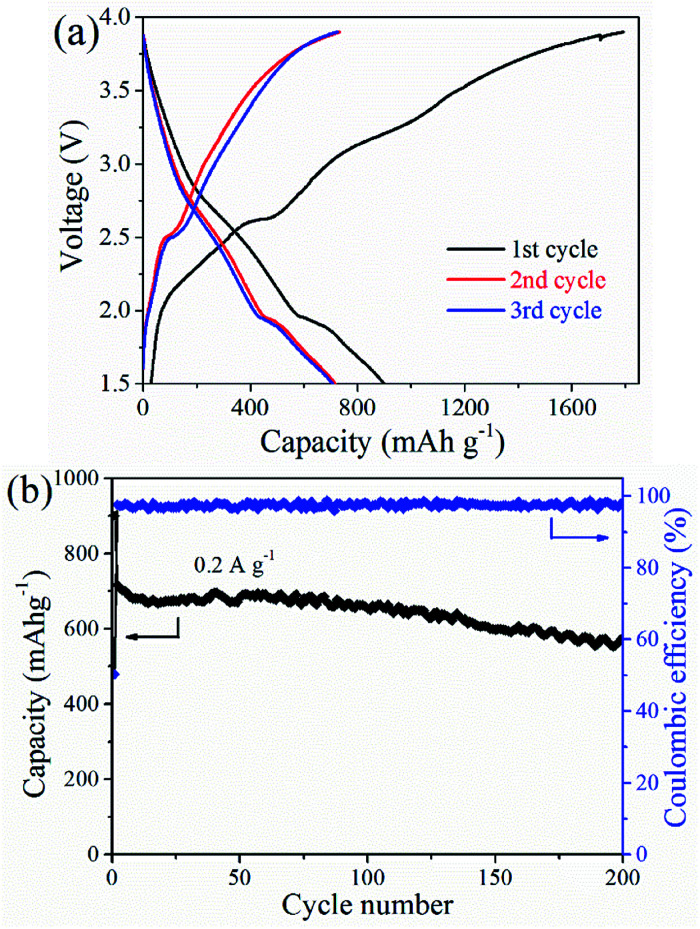
(a) Galvanostatic charge–discharge voltage profiles and (b) cycling performance of CoS@rGO-2 sample full-cell at a current density of 200 mA g^−1^.

A full cell consists of commercial LiCoO_2_ and the CoS@rGO-2 nanocomposites which respectively serve as a cathode and an anode. The capacity of the LiCoO_2_ was 1.2 times than that of the anode in design. When a current density and potential range was respectively set as 0.2 A g^−1^ and 1.5–3.9 V, the charge/discharge profiles were presented in Fig. 9a based on the CoS@rGO-2 nanocomposites as an anode. During the cycling process, charge/discharge plateaus were obviously observed at the potential range of 2.3–2.8 V and 1.6–1.8 V caused by the reversible redox reactions. The initial discharge and charge capacities calculated based on the quality of the anode materials were respectively calculated as 1791.6 and 899.6 mA h g^−1^, and the coulombic efficiency is ∼50.2%. The formation of SEI layer on the surfaces of the electrodes may account for the major loss in capacity. In the second and third cycles, the coulombic efficiency was up to 97.1% and 97.5%, respectively. This suggests that there is efficient electron transport as well as smooth insertion and extraction of Li^+^. Moreover, the cycling performance of the full battery was exhibited in Fig. 9b. A capacity maintained 574.7 mA h g^−1^ at 0.2 A g^−1^ when the battery cycled 200 times, which indicates outstanding cycling stability for the full battery.

## Conclusions

The CoS@rGO nanocomposites were synthesized through a facile solvothermal method. Some favorable electrochemical performances in specific capacity, cycling performance and rate capability are achieved when the nanocomposites were used as anode materials for LIBs. For a half-cell, the CoS@rGO-2 nanocomposite anode exhibited a capacity of 1253.9 mA h g^−1^ at 500 mA g^−1^ after 100 cycles. For a full-cell, the CoS@rGO-2 nanocomposite anode exhibited a capacity of 574.7 mA h g^−1^ at 200 mA g^−1^ after 200 cycles. In addition, in contrast to other CoS-based composite anodes reported, the as prepared nanocomposite anodes present the superior rate performance. And the CoS@rGO-2 nanocomposite delivers specific capacities of 892.1 and 668.5 mA h g^−1^, at respective 1000 mA g^−1^ and 2000 mA g^−1^. The unique nanostructure of the electrode materials accounts for the superior electrochemical behaviors. Furthermore, the rGO embedded in the nanocomposites can effectively buffer the volume variation, mitigate the aggregation of CoS particles, and prevent the CoS particles from dropping off the electrode during the cycling process. These notable electrochemical characteristics indicate promise of the nanocomposites as anode materials in LIBs.

## Conflicts of interest

There are no conflicts to declare.

## Supplementary Material

RA-010-D0RA01351J-s001

## References

[cit1] Liu L. L., Gu S. J., Wang S. L., Zhang X. Y., Chen S. M. (2020). RSC Adv..

[cit2] Huang B., Pan Z. F., Su X. Y., An L. (2018). J. Power Sources.

[cit3] Wang Q. S., Jiang L. H., Yu Y., Sun J. H. (2019). Nano Energy.

[cit4] Gao Y., Qiu X. T., Wang X. L., Chen X. C., Gu A. Q., Yu Z. L. (2020). Nanotechnology.

[cit5] Ao X., Sun H. Y., Wang C. D., Li J. G., Ruan Y. J., Li B. Z., Wu Q. H., Li Y., Jiang J. J., Yang Y. G., Mai L. Q. (2018). Carbon.

[cit6] Li R. Z., Huang J. F., Ren J. W., Cao L. Y., Li J. Y., Li B., Lu G. X., Yu A. M. (2020). J. Alloys Compd..

[cit7] Qi S. H., Xu B. L., Tiong V. T., Hu J., Ma J. M. (2020). Chem. Eng. J..

[cit8] Li Q. D., Li L., Wu P. J., Xu N., Wang L., Li M., Dai A., Amine K., Mai L. Q., Lu J. (2019). Adv. Energy Mater..

[cit9] Dong X., Deng Z. P., Huo L. H., Zhang X. F., Gao S. (2019). J. Alloys Compd..

[cit10] Camacho R.-A. P., Wu A.-M., Jin X.-Z., Dong X.-F., Li X.-N., Huang H. (2019). J. Power Sources.

[cit11] Zhao G., Cheng Y. L., Sun P. X., Ma W. X., Hao S. H., Wang X. K., Xu X. J., Xu Q. Q., Liu M. Q. (2020). Electrochim. Acta.

[cit12] Wu J. X., Ciucci F., Kim J.-K. (2020). Chem.–Eur. J..

[cit13] Wen H., Kang W. B., Liu X. G., Li W. J., Zhang L. P., Zhang C. H. (2019). RSC Adv..

[cit14] Ding X. D., Lei S., Du C. F., Xie Z. L., Li J. R., Huang X. Y. (2019). Adv. Mater. Interfaces.

[cit15] Nam J. S., Lee J. H., Hwang S. M., Kim Y. J. (2019). J. Mater. Chem. A.

[cit16] Xu Z. C., Zhang Z. Q., Li M. Y., Yin H. L., Lin H. T., Zhou J., Zhuo S. P. (2019). J. Solid State Electrochem..

[cit17] Wu J., Lau W. M., Geng D. S. (2017). Rare Met..

[cit18] Wang Y. M., Wu J. J., Tang Y. F., Lu X. J., Yang C. Y., Qin M. S., Huang F. Q., Li X., Zhang X. (2012). ACS Appl. Mater. Interfaces.

[cit19] Wang Q. H., Jiao L. F., Du H. M., Peng W. X., Han Y., Song D. W., Si Y. C., Wang Y. J., Yuan H. T. (2011). J. Mater. Chem..

[cit20] Jin R. C., Yang L. X., Li G. H., Chen G. (2015). J. Mater. Chem. A.

[cit21] Kim J. H., Lee J.-H., Kang Y. C. (2014). Electrochim. Acta.

[cit22] Wang H. J., Ma J. J., Liu S., Nie L. Y., Chai Y. Q., Yang X., Yuan R. (2016). J. Alloys Compd..

[cit23] Luo F., Ma D. T., Li Y. L., Mi H. W., Zhang P. X., Luo S. (2019). Electrochim. Acta.

[cit24] Li G. J., Mo X. Y., Law W.-C., Chan K. C. (2019). ACS Appl. Mater. Interfaces.

[cit25] Yao Y. F., Zhu Y. H., Huang J. F., Shen J. H., Li C. Z. (2018). Electrochim. Acta.

[cit26] Gao C., Fang C. Z., Zhao H. M., Yang J. Y., Gu Z. D., Sun W., Zhang W. N., Li S., Xu L. C., Li X. Y., Huo F. W. (2019). J. Power Sources.

[cit27] Huang G. C., Chen T., Wang Z., Chang K., Chen W. X. (2013). J. Power Sources.

[cit28] Li Z. P., Li W. Y., Xue H. T., Kang W. P., Yang X., Sun M. L., Tang Y. B., Lee C.-S. (2014). RSC Adv..

[cit29] Zhou Q., Liu L., Guo G. X., Yan Z. C., Tan J. L., Huang Z. F., Chen X. Y., Wang X. Y. (2015). RSC Adv..

[cit30] Tan Y. B., Liang M., Lou P. L., Cui Z. H., Guo X. X., Sun W. W., Yu X. B. (2016). ACS Appl. Mater. Interfaces.

[cit31] Zhu J. S., Ding X. B. (2019). Mater. Lett..

[cit32] Lu S.-J., Wang Z.-T., Zhang X.-H., He Z.-J., Tong H., Li Y.-J., Zheng J.-C. (2020). ACS Appl. Mater. Interfaces.

[cit33] Kong S. F., Jin Z. T., Liu H., Wang Y. (2014). J. Phys. Chem. C.

[cit34] Pei X. Y., Mo D. C., Lyu S. S., Zhang J. H., Fu Y. X. (2019). Appl. Surf. Sci..

[cit35] Sui X. Y., Huang X. K., Wu Y. P., Ren R., Pu H. H., Chang J. B., Zhou G. H., Mao S., Chen J. H. (2018). ACS Appl. Mater. Interfaces.

[cit36] Li G. J., Mo X. Y., Law W.-C., Chan K. C. (2019). J. Mater. Chem. A.

[cit37] Ma Y. H., Li Y. K., Li D., Liu Y. S., Zhang J. M. (2019). J. Alloys Compd..

[cit38] He J. R., Chen Y. F., Li P. J., Fu F., Wang Z. G., Zhang W. L. (2015). Electrochim. Acta.

[cit39] Wang X. Z., Xiao Y. H., Su D. C., Zhou L. M., Wu S. D., Han L. F., Fang S. M., Cao S. K. (2016). Electrochim. Acta.

[cit40] Wang Y. P., Zhu T., Zhang Y. F., Kong X. Z., Liang S. Q., Cao G. Z., Pan A. Q. (2017). J. Mater. Chem. A.

[cit41] Wang G. Q., Zhang J., Kuang S., Liu S. M., Zhuo S. P. (2014). J. Power Sources.

[cit42] Wang Q. F., Liang X., Yang D. P., Zhang D. H. (2017). RSC Adv..

[cit43] Yao Y. F., Zhu Y. H., Shen J. H., Yang X. L., Li C. Z. (2016). Electrochim. Acta.

[cit44] Patil S. J., Kim J. H., Lee D. W. (2017). J. Power Sources.

[cit45] Shi Q. Q., Peng F., Liao S. X., Wang H. J., Yu H., Liu Z. W., Zhang B. S., Su D. S. (2013). J. Mater. Chem. A.

[cit46] Qiao X. H., Jin J. T., Fan H. B., Li Y. W., Liao S. J. (2017). J. Mater. Chem. A.

[cit47] Ge Y. C., Wu J. J., Xu X. W., Ye M. G., Shen J. F. (2016). Int. J. Hydrogen Energy.

[cit48] Huang J. R., Wang W., Lin X. R., Gu C. P., Liu J. Y. (2018). J. Power Sources.

[cit49] Ren H. B., Wang W., Joo S. W., Sun Y. F., Gu C. P. (2019). Mater. Res. Bull..

[cit50] Zhou Q., Liu L., Guo G. X., Yan Z. C., Tan J. L., Huang Z. F., Chen X. Y., Wang X. Y. (2015). RSC Adv..

[cit51] Yang Z., Wang J., Wu H.-T., Kong F.-J., Yin W.-Y., Cheng H.-J., Tang X.-Y., Qian B., Tao S., Yi J., Ma Y.-S., Yuan R.-X. (2019). Appl. Surf. Sci..

[cit52] Zhuo L. H., Wu Y. Q., Wang L. Y., Yu Y. C., Zhang X. B., Zhao F. Y. (2012). RSC Adv..

[cit53] Endo M., Kim C., Nishimura K., Fujino T., Miyashita K. (2000). Carbon.

[cit54] Zheng Y. Y., Li Y. W., Yao J. H., Huang Y., Xiao S. H. (2018). Ceram. Int..

[cit55] Lin W. J., Huang Y. H., He G. Q. (2018). Crystengcomm.

[cit56] Gu Y., Xu Y., Wang Y. (2013). ACS Appl. Mater. Interfaces.

[cit57] Zhu J., Jin M. H., Tang S. (2019). J. Nanosci. Nanotechnol..

[cit58] Gao L. L., Gu C. P., Ren H. B., Song X. J., Huang J. R. (2018). Electrochim. Acta.

[cit59] Zhao J. J., Ren H. B., Gu C. P., Guan W. M., Song X. J., Huang J. R. (2019). J. Alloys Compd..

