# Supplementary material for: Preparation of cobalt sulfide@reduced graphene oxide nanocomposites with outstanding electrochemical behavior for lithium-ion batteries
Source: RSC Adv. 2020 Apr 2;10(23):13543–51. doi: 10.1039/d0ra01351j (PMC9051548; doi:10.1039/d0ra01351j)
Supplement: RA-010-D0RA01351J-s001 [file RA-010-D0RA01351J-s001.pdf]

Electronic Supplementary Material (ESI) for RSC Advances.
This journal is © The Royal Society of Chemistry 2020

Supplementary Material (ESI) for RSC Advances
This journal is (c) The Royal Society of Chemistry 2011

Electronic Supplementary Informations

Preparation of cobalt sulfide@reduced graphene oxide nanocomposites with outstanding electrochemical behaviors for lithium-ion batteries

Junhai Wang [a], Yongxing Zhang [b, *], Jun Wang [c], Lvlv Gao [c], Zinan Jiang [c], Haibo Ren [c], Jiarui Huang [c, *]

[a] School of Material and Chemical Engineering, Chuzhou University, Chuzhou 239000, P.R. China

[b] Anhui Province Key Laboratory of Pollutant Sensitive Materials and Environmental Remediation, Huaibei Normal University, Huaibei 235000, P.R. China

[c] Key Laboratory of Functional Molecular Solids, Ministry of Education, Key Laboratory of Electrochemical Clean Energy of Anhui Higher Education Institutes, College of Chemistry and Materials Science, Anhui Normal University, Wuhu 241002, P.R. China

Corresponding authors. E-mail: jrhuang@mail.ahnu.edu.cn; zyx07157@mail.ustc.edu.cn.

Phone. +86-553-3869-303. Fax. +86-553-3869-303.

1. Preparation of three-dimensional rGO

Modified Hummer's method was used to the prepare graphene oxide (GO). Moreover, 0.5 g of natural graphite powder was added to 15 ml of H_2SO_4 (Shanghai Aladdin Bio-Chem Technology Co., Ltd) and stirred vigorously for 20 h at room temperature, followed by the addition of 0.375 g of $NaNO_3$ (Shanghai Aladdin Bio-Chem Technology Co., Ltd) under vigorous stirring for 0.5 h with ice-treatment. Thereafter, 2.0 g of $KMnO_4$ (Shanghai Aladdin Bio-Chem Technology Co., Ltd) was gradually added to the mixed solution and stirred for 0.5 h at approximately 40 °C. The mixture was then set for 5 d at room temperature, and 60 ml deionized water was gradually added under continuous stirring for 10 min. Finally, the reaction was completed by the addition of 3 ml of 30% (weight ratio) H_2O_2 solution, resulting in the formation of a bright yellow suspension. Graphene oxide was obtained by a series of treatment processes such as centrifugation, washing with 30% HCl and deionized water, and vacuum-drying. Thereafter, the as-synthesized graphene oxide was uniformly dispersed in deionized water using ultrasound, to form a 0.9 mg ml^{-1} graphene oxide (GO) solution. The uniform GO solution was then transferred to a 25 mL Teflon-lined autoclave, and heat-treated at 230 °C for 20 h. After cooling to the room temperature, the successfully synthesized three-dimensional (3D) rGO was washed 10 times using deionized water.

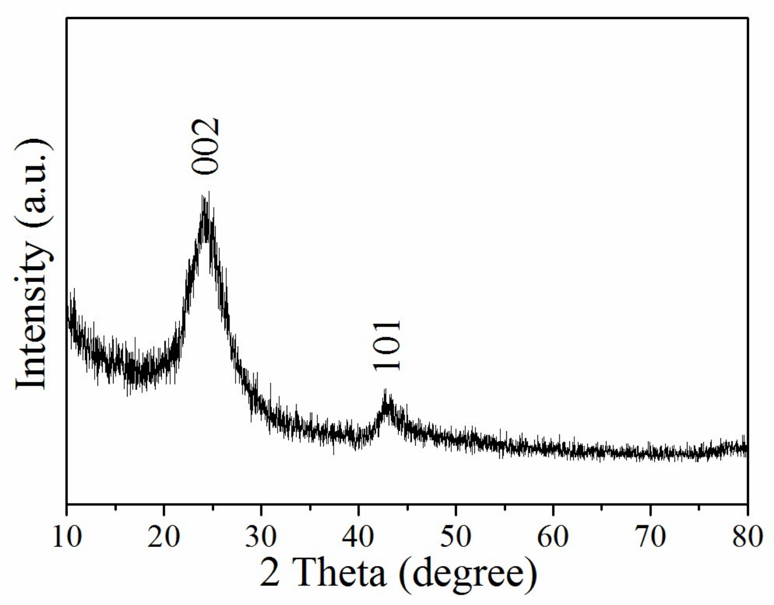

Fig. S1 XRD pattern of 3D rGO.

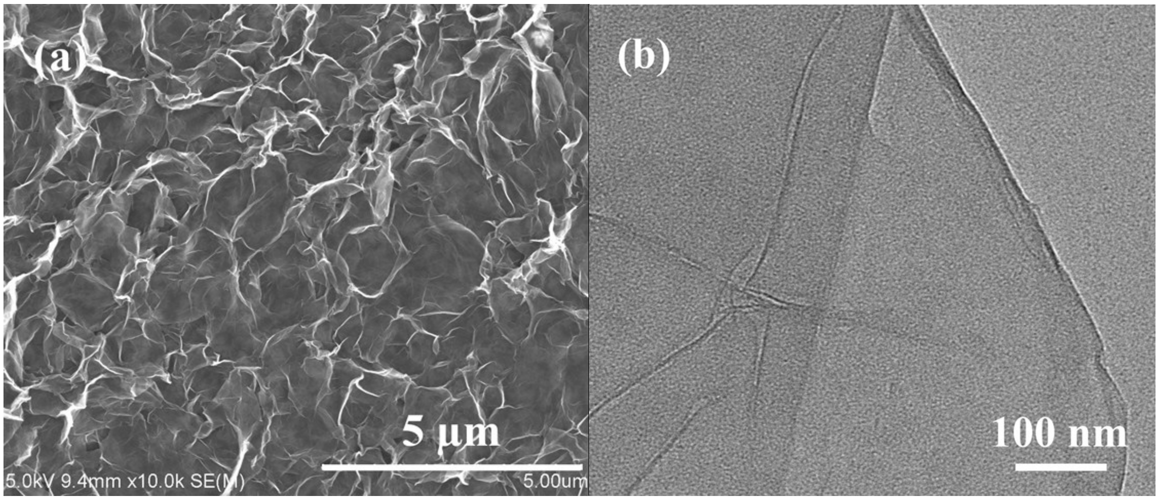

Fig. S2 (a) SEM and (b) TEM images of 3D rGO.

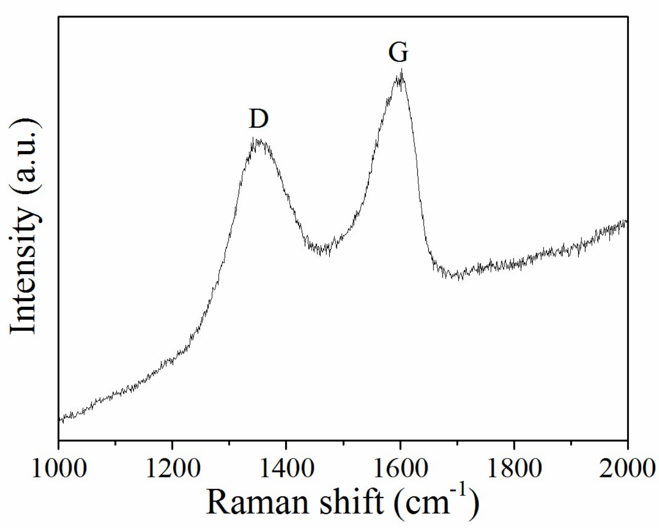

Fig. S3 Raman spectrum of GO.

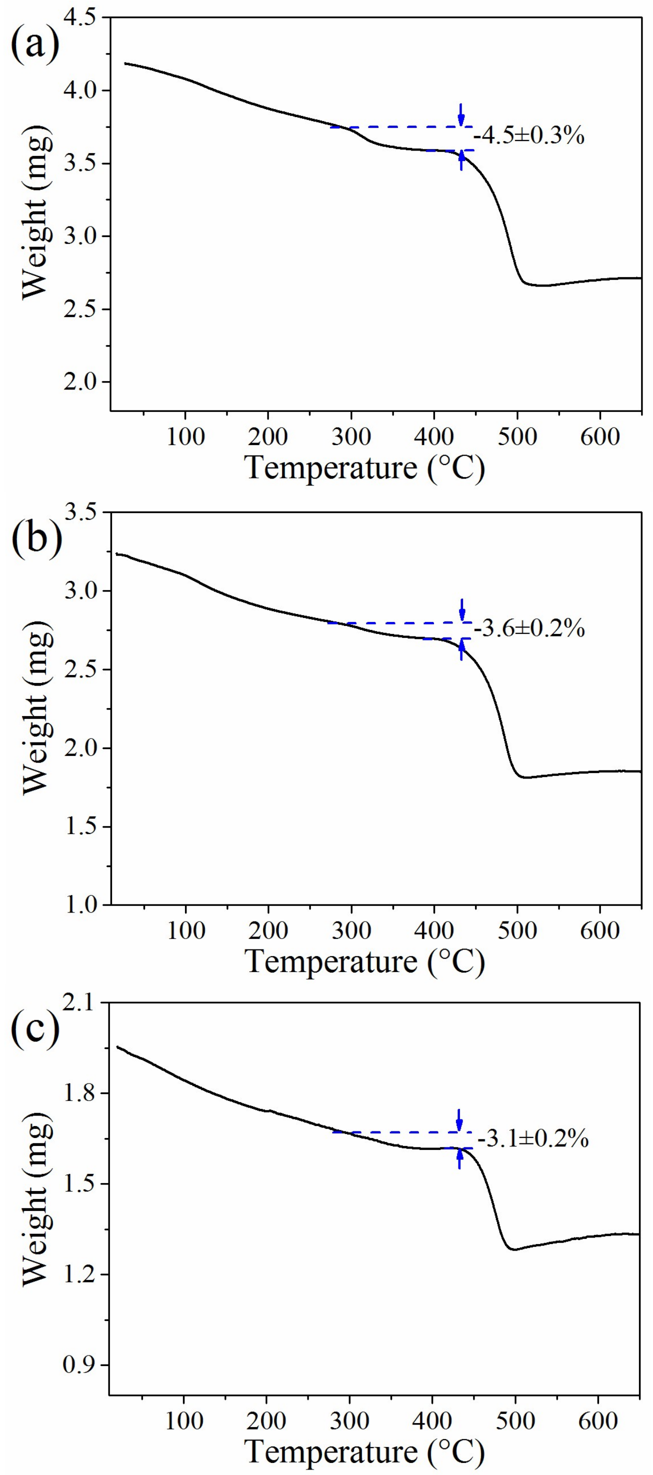

Fig. S4 TGA curves of (a) CoS@rGO-1, (b) CoS@rGO-2 and (c) CoS@rGO-3 composites from 18 to 650 °C in air at a heating rate of 10 °C min^{-1}.

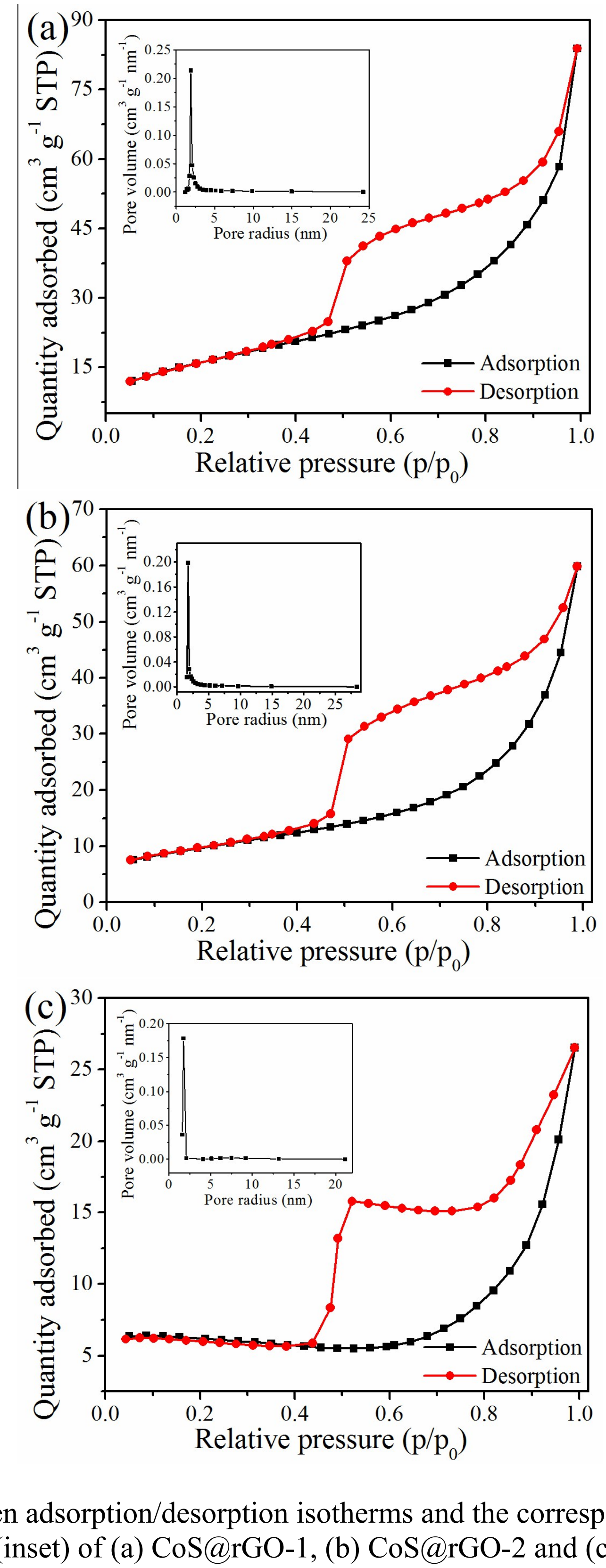

Fig. S5 Nitrogen adsorption/desorption isotherms and the corresponding pore size distributions (inset) of (a) CoS@rGO-1, (b) CoS@rGO-2 and (c) CoS@rGO-3 composites.

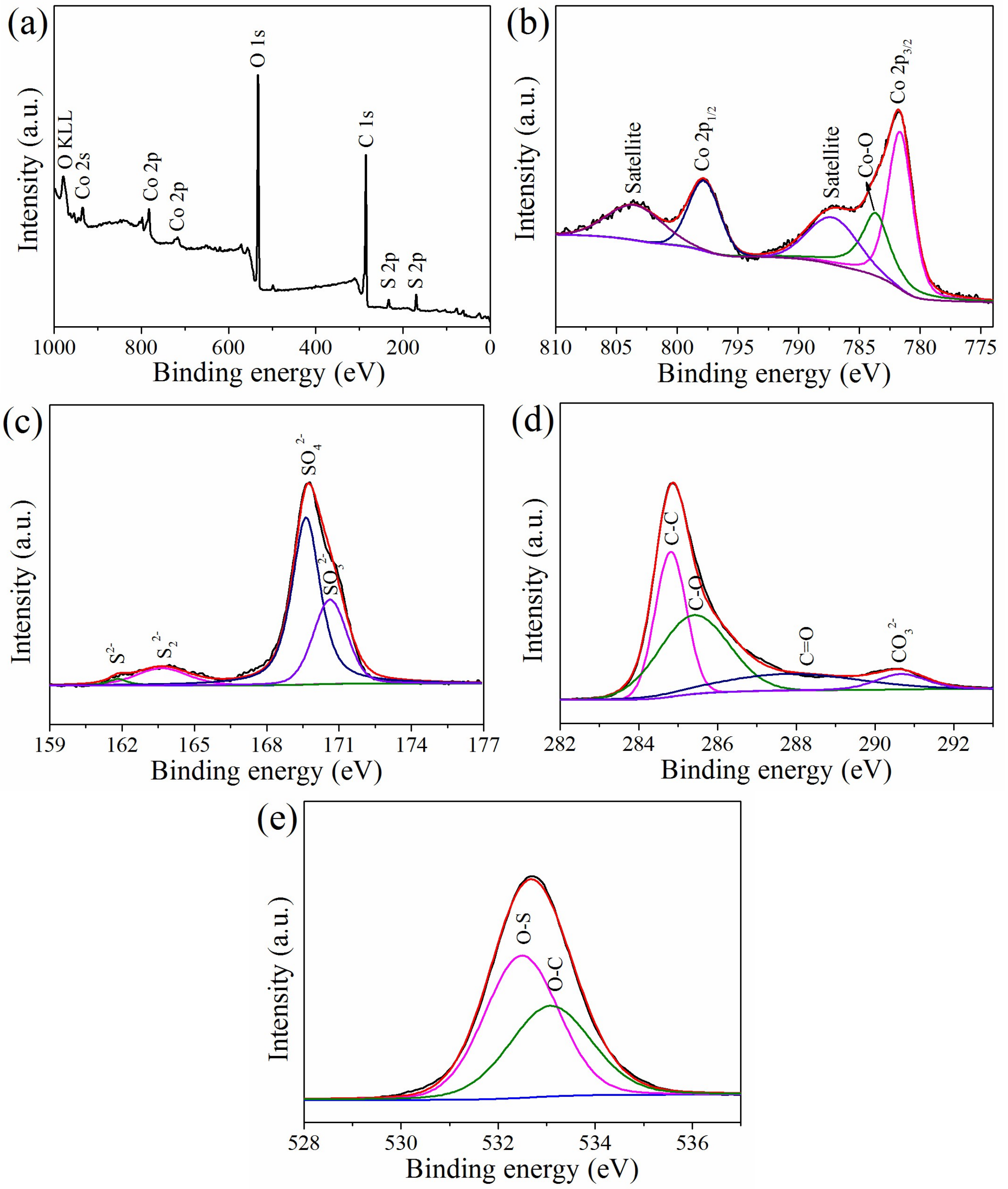

Fig. S6 XPS spectra of CoS@rGO-2 sample after 100 cycles: (a) survey spectrum, (b) Co 2p spectrum, (c) S 2p spectrum, (d) C 1s spectrum, and (e) O 1s spectrum.

Table S1 Comparison of electrochemical properties of CoS_x-related electrodes.

Electrode material	Reversible capacity	Cycle times	Current density	Ref.
CoS NPs	589 mAh g^{-1}	10	0.1 A g^{-1}	[18]
Lantern-like CoS	477 mAh g^{-1}	400	0.1 A g^{-1}	[55]
CoS/CNTs hybrid	780 mAh g^{-1}	50	0.1 A g^{-1}	[22]
CoS nanosheets/rGO foams	481.7 mAh g^{-1}	100	0.06 A g^{-1}	[25]
Cobalt sulfides/rGO composite	950 mAh g^{-1}	50	0.1 A g^{-1}	[27]
Cobalt sulfide/rGO composite	994 mAh g^{-1}	150	0.2 A g^{-1}	[28]
CoS NFs-rGO	939 mAh g^{-1}	100	0.1 A g^{-1}	[30]
CoSx/rGO nanocomposite	796 mAh g^{-1}	50	0.1 A g^{-1}	[31]
$Co_{1-x}S$ hollow spheres/rGO	969.8 mAh g^{-1}	90	0.05 A g^{-1}	[32]
CoS nanosheets/rGO	898 mAh g^{-1}	80	0.0589 A g^{-1}	[33]
rGO-wrapped CoS NPs	749 mAh g^{-1}	40	0.0625 A g^{-1}	[56]
CoS NPs/graphene sheets	600 mAh g^{-1}	200	0.1 A g^{-1}	[57]
CoS_2/rGO composite	831 mAh g^{-1}	300	1.0 A g^{-1}	[38]
CoS@rGO-1	868.1 mAh g^{-1}	100	0.5 A g^{-1}	(This work)
CoS@rGO-2	1253.9 mAh g^{-1}	100	0.5 A g^{-1}	(This work)
CoS@rGO-3	1056.6 mAh g^{-1}	100	0.5 A g^{-1}	(This work)

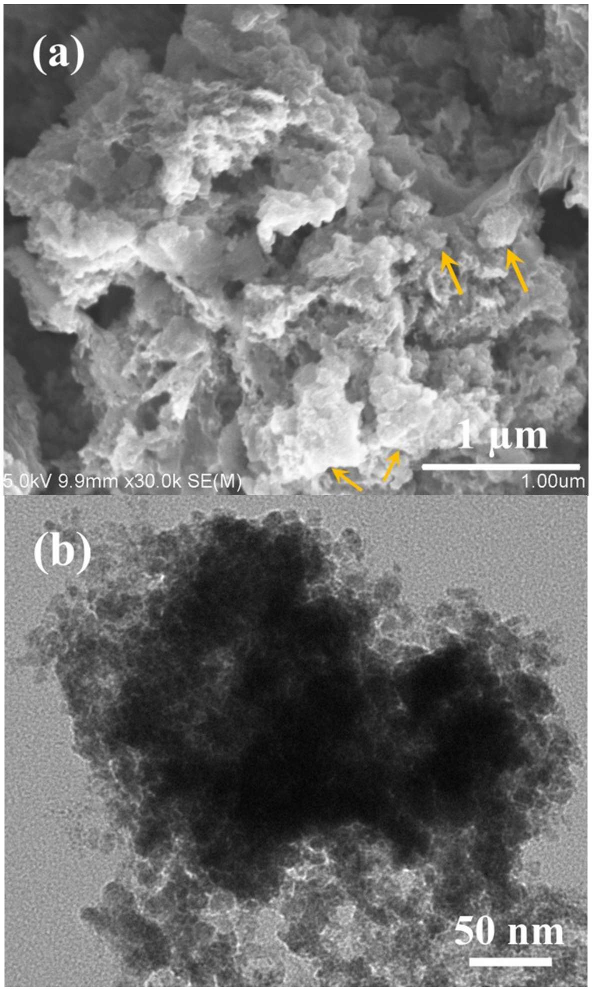

Fig. S7 (a) SEM image and (b) TEM image of CoS@rGO-3 electrode after 100 cycles at a current density of 500 mA g^{-1}.

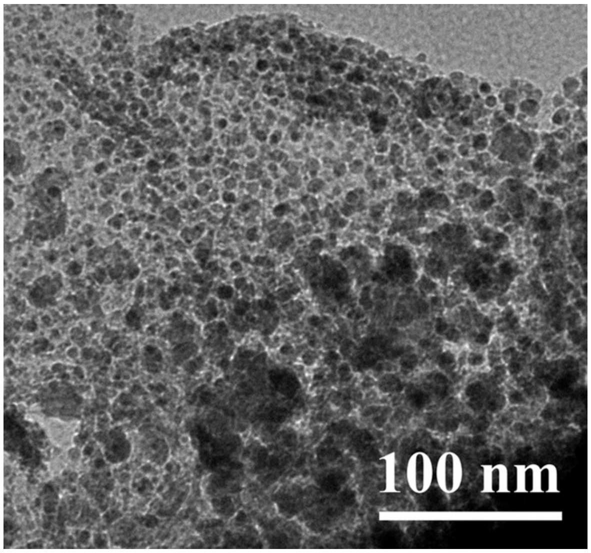

Fig. S8 TEM image of CoS@rGO-2 electrode after 10 cycles at a current density of 500 mA g^{-1}.
